# *CYP11A1* Upregulation Leads to Trophoblast Oxidative Stress and Fetal Neurodevelopmental Toxicity That can be Rescued by Vitamin D

**DOI:** 10.3389/fmolb.2020.608447

**Published:** 2021-02-15

**Authors:** Xiang Wang, Mengxue Li, Xueguang Zhang, Yaqian Li, Guolin He, Andras Dinnyés, Qun Sun, Wenming Xu

**Affiliations:** ^1^ Department of Obstetrics/Gynecology, Joint Laboratory of Reproductive Medicine (SCU-CUHK), Key Laboratory of Obstetric, Gynecologic and Pediatric Diseases and Birth Defects of Ministry of Education, West China Second University Hospital, Sichuan University, Chengdu, China; ^2^ Reproductive Endocrinology and Regulation Laboratory, West China Second University Hospital, Sichuan University, Chengdu, China; ^3^ Key Laboratory of Bio-resources and Eco-environment of the Ministry of Education, College of Life Sciences, Sichuan University, Chengdu, China; ^4^ Department of Obstetrics and Gynecology, Key Laboratory of Birth Defects and Related Disease of Women and Children, Ministry of Education, West China Second University Hospital, Sichuan University, GödöllőChengdu, Hungary; ^5^ BioTalentum Ltd.,, Gödöllő, Hungary

**Keywords:** CYP11A1, P450scc, neuronal stem cells, trophoblast, vitamin D

## Abstract

During normal pregnancy, the placental trophoblast secretes a variety of steroid hormones and participates in the regulation of maternal physiological functions and fetal development. The *CYP11A1* gene encodes the cholesterol side-chain cleavage enzyme P450scc, which catalyzes the production of pregnenolone from cholesterol, which is the first step in the synthesis of all steroid hormones. Under the influence of genetic susceptibility and certain environmental factors, such as drugs and toxins, the expression of *CYP11A1* can be upregulated, thereby affecting steroid metabolism and physiological functions in trophoblast cells, as well as fetal development. Here*,* we demonstrate that upregulation of *CYP11A1* in the BeWo cell line triggers excessive mitochondrial oxidative stress, leads to mitochondrial damage and interleukin-6 release, and contributes to the inhibition of proliferation and DNA damage in neuronal stem cells (NSCs). Furthermore, oxidative stress and inflammation can be ameliorated by vitamin D_3_ in a dose-dependent manner, thereby facilitating the rescue of NSC impairment. Our findings reveal the underlying mechanism in which upregulation of *CYP11A1* is detrimental to the physiological function of trophoblasts and demonstrate the beneficial effects of vitamin D supplementation in preventing placental and neurodevelopmental damage associated with *CYP11A1* upregulation during pregnancy.

## Introduction

In humans, the *CYP11A1* gene is located on chromosome 15, and it encodes a cholesterol side-chain cleavage enzyme, P450scc, that is highly expressed in organs associated with the synthesis of steroid hormones, such as the adrenal gland, ovary, testis, and placenta (Hanukoglu, 1992; Strauss et al., 1996). P450scc catalyzes the production of pregnenolone from cholesterol, which is the rate-limiting step in steroidogenesis (Churchill and Kimura, 1979; Hanukoglu et al., 1990; Hanukoglu, 1992). Unlike the adrenal gland, ovary, and testis, the placenta does not express steroidogenic acute regulatory protein (StAR), which is responsible for transportation of cholesterol to the mitochondrial inner membrane; this is the real reason for the rate limitation in the afore mentioned organs (Stocco, 1999; Christenson and Strauss, 2000; Miller, 2007). Tuckey et al. pointed out that the supply of cholesterol to P450scc may approach saturation, and this rate limitation may be associated with the inhibition of P450scc activity by adrenodoxin reductase and its products (Tuckey, 2005). Thus, regulating *CYP11A1* expression levels in the placental trophoblast can, theoretically, change steroidogenesis, thereby affecting the mitochondrial function and metabolic behavior of trophoblasts.

It has been demonstrated that a variety of factors in the intrauterine environment, such as drugs, toxins, hormones, and inflammation, can upregulate *CYP11A1* expression in the trophoblast cells and impair pregnancy outcome (Beaudoin et al., 1997; Wang et al., 2014; Zhang et al., 2016b; Zhang et al., 2019). *CYP11A1* has been identified as a candidate pathogenic gene for preeclampsia and shown to be hypomethylated and transcribed at higher levels in women with preeclampsia (Enquobahrie et al., 2008; Hogg et al., 2013). Our previous studies have demonstrated that upregulation of *CYP11A1* induces trophoblast apoptosis and is positively correlated with liver damage during preeclampsia; however, the underlying mechanism remains elusive (He et al., 2013; Pan et al., 2017). In addition, due to the essential roles of steroid hormones in pregnancy, a valid question is whether or not the upregulation of this key gene has other negative effects.

Vitamin D is a vital nutrient for the maintenance of calcium homeostasis. In contrast to typical metabolism, in which vitamin D is first hydroxylated to calcidiol (25(OH)D) by CYP2R1 in the liver and then further hydroxylated to calcitriol (1,25(OH)D) by CYP27B1 in the kidney, Guryev (Guryev et al., 2003) and Zbytek et al (Zbytek et al., 2008). have uncovered a novel metabolic pathway, in which vitamin D is catalyzed to 20(OH)D by CYP11A1 (P450scc) in the placenta and showed anti-inflammatory activity and differential regulation (Guryev et al., 2003). In recent years, accumulating evidence from studies on pregnant women has demonstrated the preventive and therapeutic effects of vitamin D supplementation during pregnancy, especially in preventing preeclampsia. Fogacci et al. reviewed 27 clinical trials focusing on vitamin D supplementation and preeclampsia and concluded that although the underlying mechanism was unclear and the timing of dosage was controversial, the preventive effect of vitamin D supplementation on preeclampsia was firmly established (Fogacci et al., 2020). Based on these findings, we hypothesized that vitamin D could affect the impairment caused by *CYP11A1* upregulation and that the underlying mechanisms may involve competitive binding to excess P450scc to decrease steroidogenesis and promote anti-inflammation via 20(OH)D production.

In this study, the BeWo cell line, a well-established *in vitro* trophoblast model for studying placental endocrine function, was used to investigate the signaling cascade triggered by *CYP11A1* overexpression, including excessive oxidative stress, DNA damage, and apoptosis of mitochondria, leading to inflammatory cytokine release and contributing to inhibition of proliferation and DNA damage in neuronal stem cells (NSCs). In addition, we studied the putative role of vitamin D_3_ in preventing these negative effects. Overall, this study may provide a theoretical basis for the application of vitamin D supplementation during pregnancy.

## Results

### *CYP11A1* overexpression in the BeWo cell line

**FIGURE 1:**
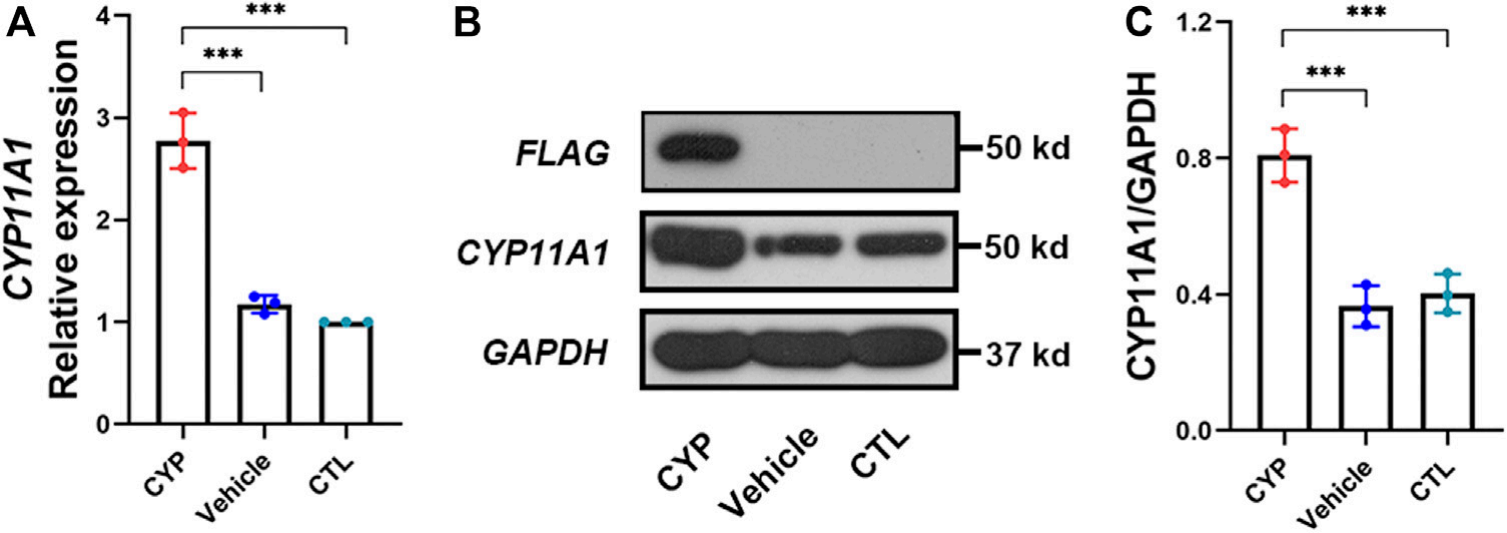
*CYP11A1*expression in BeWo cells was significantly increased after lentivirus transfection. **(A)** Relative mRNA expression levels and **(B, C)** protein expression levels of *CYP11A1*. CYP, BeWo cells overexpressing *CYP11A1*. Vehicle, BeWo cells transfected with vehicle. CTL, control. Statistical analysis was performed on the means from three independent experiments using one-way analysis of variance (ANOVA) with Tukey’s post hoc test. ***P < 0.001.

After overexpression of *CYP11A1* in BeWo cells and selection using puromycin, expression of the *CYP11A1* gene was determined at the mRNA and protein level using qPCR and western blotting, respectively. Results showed significantly increased levels of *CYP11A1* mRNA and protein compared to those in the vehicle or control cells (Figure 1). Interestingly, we also observed upregulation of *CYP11A1* with low-dose bisphenol A (BPA) treatment (Supplemental Figure S1), indicating that both genetic variants and environmental factors, such as BPA, may lead to *CYP11A1* upregulation in the placenta.

### CYP11A1 Overexpression in BeWo Cells Induced Excessive Mitochondrial Biosynthesis That Could Be Ameliorated by Vitamin D_3_

**FIGURE 2:**
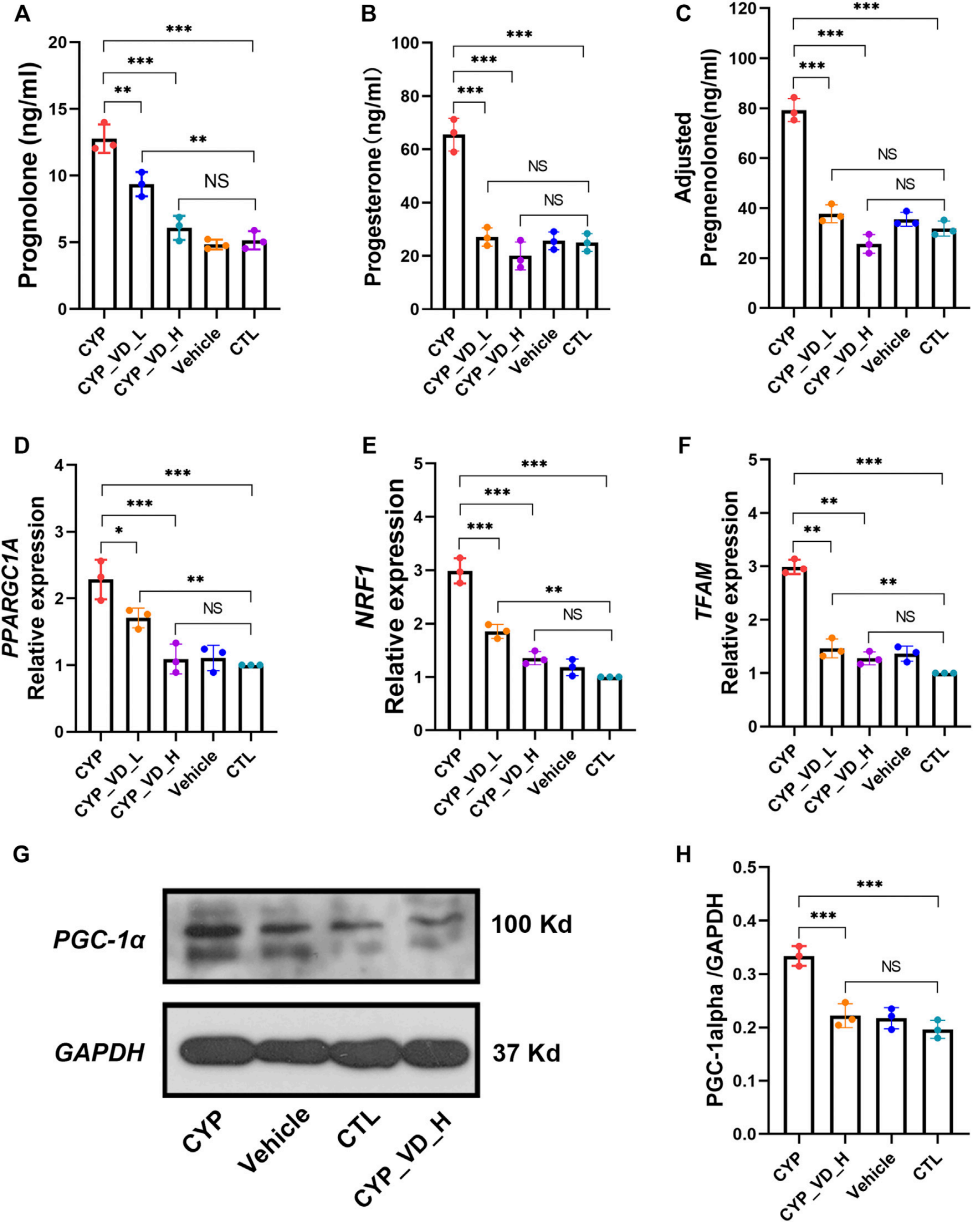
Excessive mitochondrial biosynthesis induced by upregulation of *CYP11A1* in BeWo cells could be ameliorated by vitamin D_3_ addition. **(A–C)** Pregnenolone and progesterone secretion. **(D–F)** Relative expression of mitochondrial biosynthesis markers at the mRNA level. (G, H) Relative expression of PGC1-α. CYP, BeWo cells overexpressing *CYP11A1*. Vehicle, BeWo cells transfected with vehicle. CTL, control. CYP_VD_L, BeWo cells overexpressing *CYP11A1* and treated with a low-dose of vitamin D_3_ (2 μM). CYP_VD_H, BeWo cells overexpressing *CYP11A1* and treated with a high-dose of vitamin D_3_ (5 μM). Statistical analysis was performed on the means from three independent experiments using one-way analysis of variance (ANOVA) with Tukey’s post hoc test. *P < 0.05, **P < 0.01, ***P < 0.001; NS, no significance.

First, we analyzed the secretion of pregnenolone, a direct product of catalysis by P450scc. Pregnenolone production increased significantly with *CYP11A1* overexpression and was reduced by the addition of 2 μM or 5 μM vitamin D_3_ (Figure 2A). Furthermore, the same trend was also observed for the secretion of progesterone, an indirect product of P450scc activity (Figure 2B). It should be noted that in trophoblast cells, progesterone is only catalyzed from pregnenolone by mitochondrial 3β-hydroxysteroid dehydrogenase type I (3βHSD-I), therefore, total levels of secreted pregnenolone should equal at least the sum of detected pregnenolone and progesterone (Figure 2C).

Secretion of pregnenolone and progesterone requires the support of the mitochondrial biosynthesis system. Next, we observed a marked increase in the expression of the mitochondrial biosynthesis markers, namely *PPARGC1A*, *NRF1*, and *TFAM* at the mRNA level*. PPARGC1A* encodes PGC1-α, a member of the transcriptional coactivators and the master regulator of mitochondrial biogenesis. PGC1-α activates *NRF1*, which in turn co-activates *TFAM*, which is responsible for transcribing nuclear-encoded mitochondrial proteins. This alteration could be decreased and rescued by 2 μM and 5 μM of vitamin D_3_, respectively, (Figures 2D–F). Expression of these mitochondrial biosynthesis markers, represented by *PPARGC1A*, at the protein level was determined using western blotting, and displayed the same trend as described above (Figures 2G,H).

Together, these results indicated that the direct effect of CYP11A1 upregulation was excessive mitochondrial biosynthesis, which could be rescued by the addition of vitamin D3.

### CYP11A1 Overexpression in BeWo Cells Resulted in Excessive Mitochondrial Reactive Oxygen Species Generation and Mitochondrial DNA Damage That Can Be Rescued by Vitamin D_3_

**FIGURE 3:**
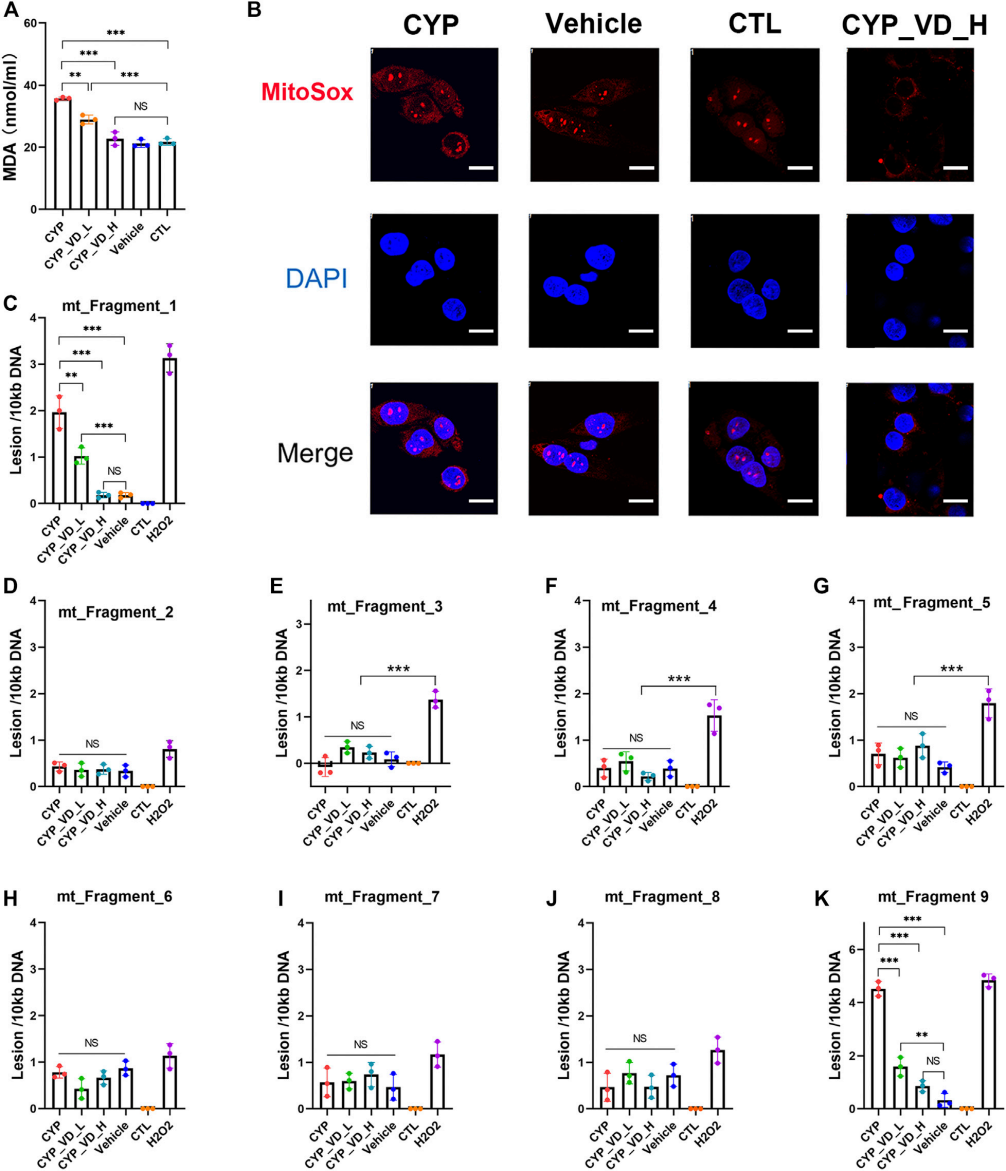
ROS accumulation and mtDNA damage induced by *CYP11A1* overexpression could be rescued by vitamin D_3_in BeWo cells. **(A)** MDA production. **(B)** Mitochondrial ROS (red fluorescence). **(C–K)** mtDNA damage induced by ROS. CYP, BeWo cells overexpressing *CYP11A1*. Vehicle, BeWo cells transfected with vehicle. CTL, control. CYP_VD_L, BeWo cells overexpressing *CYP11A1* and treated with a low-dose of vitamin D_3_ (2 μM). CYP_VD_H, BeWo cells overexpressing *CYP11A1* and treated with a high-dose of vitamin D_3_ (5 μM). Statistical analysis was performed on the means from three independent experiments using one-way analysis of variance (ANOVA) with Tukey’s post hoc test. *P < 0.05, **P < 0.01, ***P < 0.001; NS, no significance. Scale bars, 50 μm.

To evaluate whether excessive mitochondrial biosynthesis would lead to increased levels of reactive oxygen species, we analyzed levels of malonaldehyde (MDA), a product of lipid peroxidation generated by ROS. Results showed that overexpression of *CYP11A1* induced a significant increase in MDA levels that could be decreased and rescued by 2 μM and 5 μM vitamin D_3_, respectively, (Figure 3A). Then, we searched for the origin of the excessive ROS. Although most intracellular ROS are produced in the mitochondria, they can also be detected in other cell organelles and in the cytoplasm. To determine the level of ROS specifically produced by mitochondria, we used a MitoSOX™ Red mitochondrial ROS probe, which has higher selectivity and specificity for ROS in mitochondria compared with other ROS probes such as DCFH-DA. As expected, higher ROS levels (higher red fluorescence signals) were observed in the *CYP11A1* overexpression group compared to those in the vehicle or control groups (Figure 3B). In addition, treatment with 5 μM vitamin D_3_ significantly reduced ROS levels (Figure 3B).

Intracellular ROS is known to attack lipids and frequently damage mtDNA. The level of mtDNA damage was determined using qPCR, which demonstrated a significantly higher rate of DNA lesions within fragment 1 and fragment 9 (ChrM: 15361–2036) in the *CYP11A1* overexpression group. The level of mtDNA damage was similar to that observed in cells treated with 500 μM H_2_O_2_. In contrast, no obvious damage was observed in fragments 3–8, indicating that the mtDNA damage was likely caused by ROS. Following treatment with 2 μM and 5 μM vitamin D_3_, the lesion rates in fragment 1 and fragment 9 gradually decreased (Figures 3C–K).

In brief, excessive mitochondrial biosynthesis in BeWo cells induced by *CYP11A1* overexpression could lead to abnormally high mitochondrial ROS levels, manifested by increased MDA content and MitoSOX fluorescence signals, and hence damaged the mtDNA. Furthermore, addition of vitamin D_3_ decreased the ROS levels.

### CYP11A1 Overexpression in BeWo Cells Caused Mitochondrial Apoptosis and Inflammation in the Trophoblast

**FIGURE 4:**
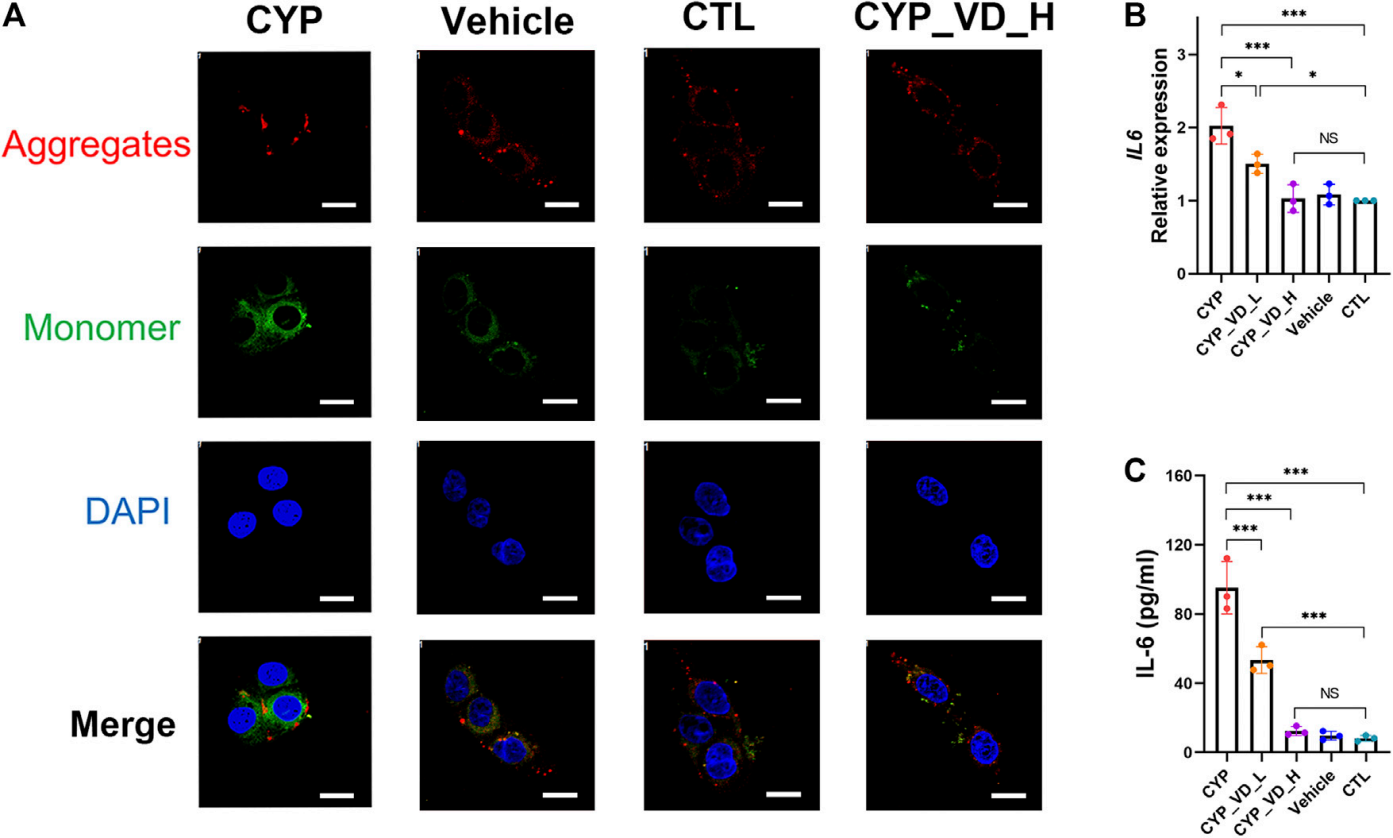
Trophoblast mitochondrial apoptosis and inflammation induced by upregulation of *CYP11A1* could be ameliorated by vitamin D_3_addition. **(A)** JC-1 staining of BeWo mitochondria. Red fluorescence represents JC-1 aggregates, whereas green fluorescence represents JC-1 monomers. **(B)** Transcriptional levels of IL-6 in BeWo cells. **(C)** IL-6 released by BeWo cells. CYP, BeWo cells overexpressing *CYP11A1*. Vehicle, BeWo cells transfected with vehicle. CTL, control. CYP_VD_L, BeWo cells overexpressing *CYP11A1* and treated with a low-dose of vitamin D_3_ (2 μM). CYP_VD_H, BeWo cells overexpressing *CYP11A1* and treated with a high-dose of vitamin D_3_ (5 μM). Statistical analysis was performed on the means from three independent experiments using one-way analysis of variance (ANOVA) with Tukey’s post hoc test. *P < 0.05, **P < 0.01, ***P < 0.001; NS, no significance. Scale bars, 50 μm.

Mitochondrial apoptosis occurs frequently, along with ROS and mtDNA damage, and can be assessed by checking the membrane potential. Results of JC-1 staining revealed significantly decreased levels of JC-1 aggregates (red fluorescence) and increased levels of JC-1 monomers (green fluorescence) in the *CYP11A1* overexpression group compared to those in vehicle and control groups, indicating a reversed mitochondrial membrane potential. This tendency to undergo apoptosis was rescued by treatment with 5 μM vitamin D_3_ (Figure 4A).

To evaluate the effects of mitochondrial functional impairment on the trophoblast itself, we tested BeWo cells with an inflammatory cytokine panel that included interleukin (IL)-1β, IL-4, IL-6, IL-8, IL-10, IL-13, IL-17α, and IL-23, and tumor necrosis factor α (TNF-α) using a Luminex^TM^ multiplex suspension array. Results showed that only IL-6 was present in the cells (data not shown). We further verified the production of IL-6 using qPCR and ELISA. Strikingly, the alteration in the inflammatory status of BeWo cells was verified. Furthermore, the expression and release of IL-6 was significantly increased in the *CYP11A1* overexpression group compared to the vehicle or control groups. Moreover, after adding vitamin D_3_, the inflammation gradually decreased to regular levels (Figures 4B,C).

In this part, we examined adverse effects including mitochondrial apoptosis and cellular inflammation alterations, probably caused by increased mitochondrial ROS levels and subsequent mtDNA damage. The results showed that upregulation of *CYP11A1* in BeWo cells may finally impair BeWo mitochondria and lead to inflammatory cytokines release. Addition of vitamin D_3_ rescued BeWo cells from these adverse effects.

### Impaired trophoblast exposure inhibits NSC proliferation and causes damage to genomic DNA

**FIGURE 5:**
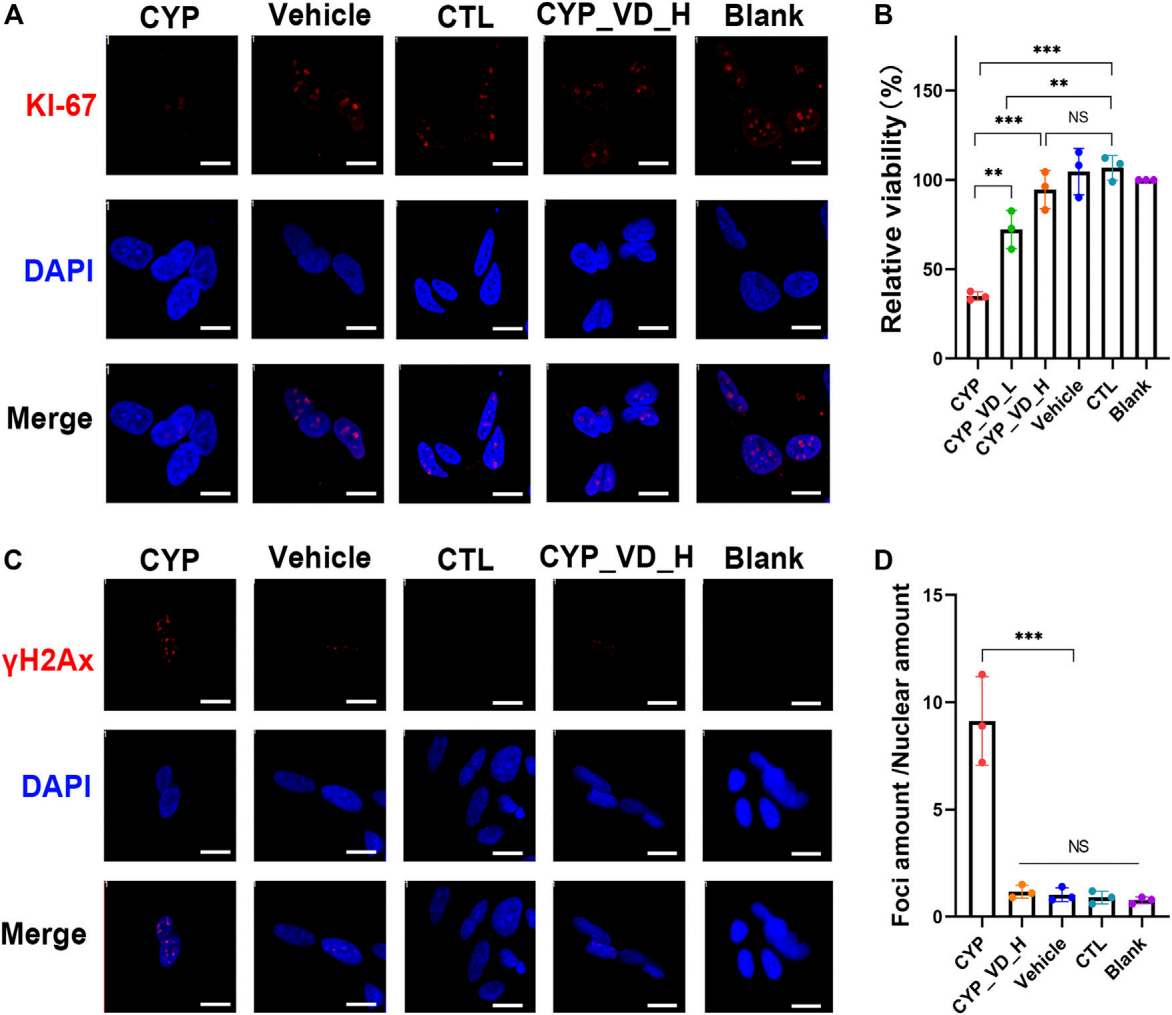
Inhibition of NSC proliferation and DNA damage induced by the conditioned medium of BeWo cells overexpressing *CYP11A1* and intervention by vitamin D_3_. **(A)** KI-67 immunostaining in NSCs. **(B)** CCK-8 proliferation assay. **(C, D)** γH2AX immunostaining in NSCs. CYP, BeWo cells overexpressing *CYP11A1*. Vehicle, BeWo cells transfected with vehicle. CTL, control. CYP_VD_L, BeWo cells overexpressing *CYP11A1* and treated with a low-dose of vitamin D_3_ (2 μM). CYP_VD_H, BeWo cells overexpressing *CYP11A1* and treated with a high-dose of vitamin D_3_ (5 μM). Statistical analysis was performed on the means from three independent experiments using one-way analysis of variance (ANOVA) with Tukey’s post hoc test. ***P < 0.001. Scale bars, 20 μm.

To investigate the effects of the inhibition of proliferation and DNA damage on NSCs, NSCs were cultured for 12 h with neuronal maintenance medium (NMM) preconditioned by BeWo cells overexpressing *CYP11A1* and with vitamin D_3_ treated. KI-67 immunostaining, which labels cells in the active phases of the cell cycle (G1, S, G2, and M phases), revealed a marked inhibition in proliferation of NSCs exposed to *CYP11A1*-upregulated BeWo cells; the inhibition of proliferation could be rescued by treatment with vitamin D_3_ (Figure 5A). This result was verified using the Cell Counting Kit-8 (CCK-8) assay (Figure 5B).

To assess DNA damage in NSCs, we performed γH2AX immunofluorescence assays. When double-strand breaks (DSBs) occur in DNA, phosphorylation of H2AX at serine 139 is induced, resulting in the production of γH2AX. Immunofluorescence staining using anti-γH2AX antibodies reveals the formation of distinct foci around DSBs. Our results showed a highly significant increase in the quantity of γH2AX foci in NSCs exposed to BeWo cells expressing *CYP11A1*, indicating increased levels of DNA damage in these NSCs. Notably, vitamin D_3_ treatment effectively rescued this damage (Figures 5C,D).

In brief, here we try to extrapolate the adverse effects induced by overexpression of *CYP11A1* in BeWo cells through culturing NSCs in BeWo conditioned medium. Results showed that the proliferation of NSCs was inhibited and DNA damage was observed. By adding vitamin D3 during medium conditioning, the damage to NSCs could be reduced.

## Discussion

In this study, we demonstrated that the overexpression of *CYP11A1* in trophoblasts increased mitochondrial steroid synthesis resulting in a significant increase in the secretion of the direct product pregnenolone, as well as the indirect product progesterone. To support these metabolic changes, biosynthesis in mitochondria is passively increased. The observed increase in the expression levels of mitochondrial biosynthesis markers *PPARGC1A*, *NRF1*, and *TFAM* further supports this mechanism. Because of their vital role in transportation and hormone synthesis during pregnancy, the mitochondrial copy number and biosynthetic activities are originally higher in trophoblasts than in many other tissues (Miller, 2013; Martinez et al., 2015). Therefore, further increasing the mitochondrial activity inevitably leads to various impairments, such as ROS accumulation and consequent mtDNA damage. In this study, the damage to mtDNA caused by ROS was most distinct in ChrM: 15361–369, which harbors most of the mitochondrial D-loop (ChrM: 16024–576). This result is in agreement with those of previous studies (Rothfuss et al., 2010). The promoter of the heavy and light strands of the mitochondrial genome is located in this region (Sharma et al., 2005; Barshad et al., 2018), indicating that this damage can significantly affect mitochondrial function and cellular metabolic behavior, including mitochondrial genomic mutation, mitochondrial autophagy, and apoptosis. These findings further explain the underlying mechanism of trophoblast cell autophagy and apoptosis, triggered by the upregulation of *CYP11A1* (Pan et al., 2017), and contribute to our understanding of the association between *CYP11A1* and preeclampsia.

**FIGURE 6:**
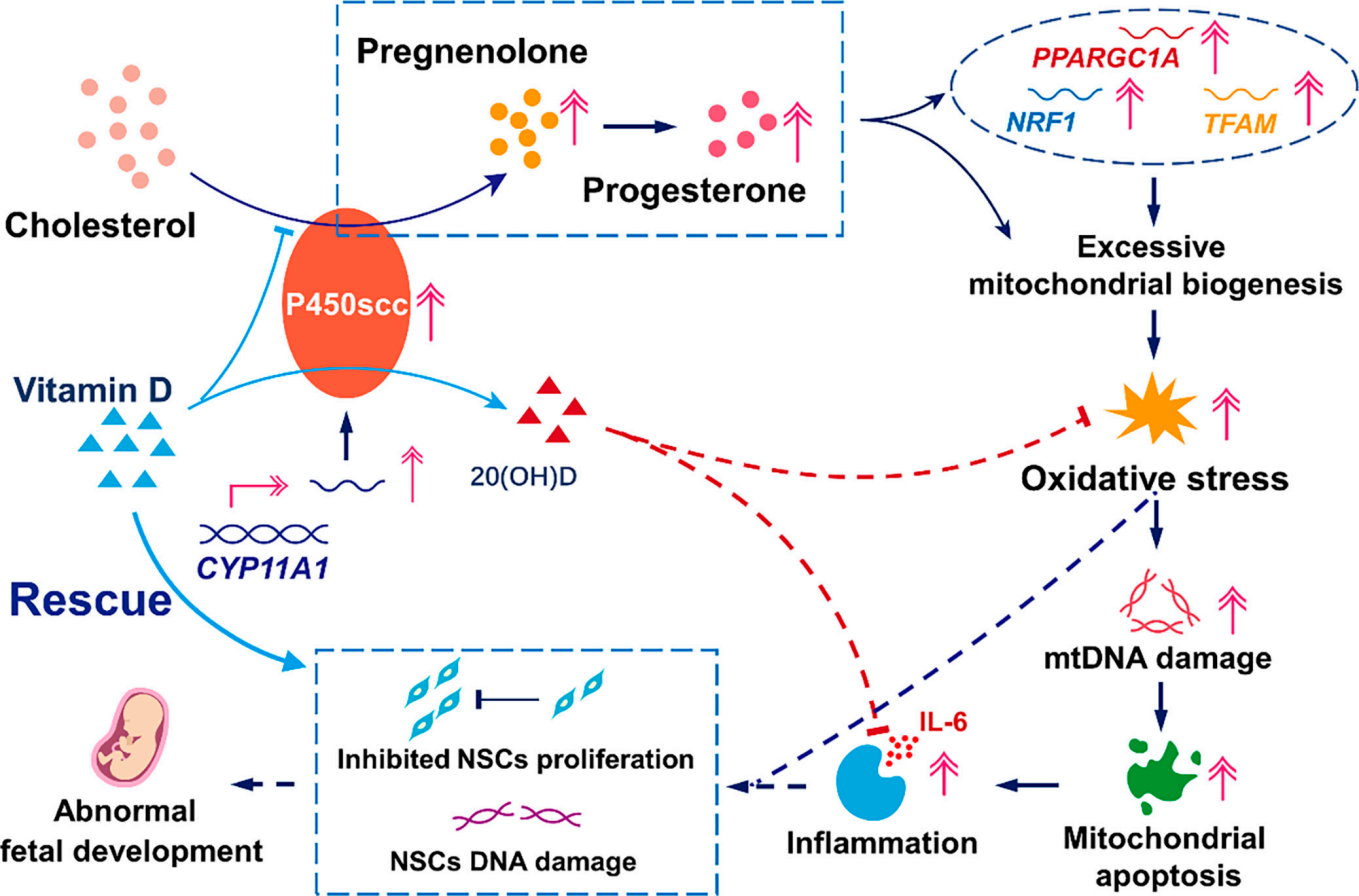
Model mechanism of the effects of upregulation of *CYP11A1* in BeWo cells and amelioration of these effects by Vitamin D. The upregulation of *CYP11A1* resulted in excessive mitochondrial biosynthesis, which triggered oxidative stress and led to trophoblast mitochondrial apoptosis and inflammation. This functional change in trophoblasts led to impaired NSCs characterized by inhibited proliferation and increased levels of DNA damage. Vitamin D supplementation proved to be an efficient intervention for the effects of *CYP11A1* upregulation, mainly via its competitive binding to P450scc and the anti-inflammatory effect due to the subsequent production of 20(OH)D.

Although the pathogenesis of preeclampsia has not been fully elucidated, it is thought that inflammation plays a central role (Wolf et al., 2001; LaMarca et al., 2007; Ruma et al., 2008; Harmon et al., 2016). Previous studies have shown that autophagy, apoptosis, and necrosis occur in trophoblast cells and that secretion of inflammatory factors, especially IL-6, increases dramatically in preeclampsia (Mulla et al., 2009; Guinazu et al., 2012; Hawkins et al., 2018). In our study, although levels of other inflammatory cytokines were not altered, IL-6 increased over nine times in cells overexpressing *CYP11A1* compared to the control cells, indicating that IL-6 expression is specifically induced by *CYP11A1* overexpression in trophoblasts. In addition to preeclampsia, inflammation in trophoblasts has been associated with fetal developmental abnormalities like intrauterine growth restriction and neurodevelopmental disorders, including neurodevelopmental delay, cognitive impairment, and autism (Challis et al., 2009; Boutsikou et al., 2010; Goeden et al., 2016; Osokine and Erlebacher, 2017; Tang et al., 2017; Rudolph et al., 2018). A recent report indicated that inflammation was the mediator of the effect of any prenatal environmental adversity on fetal neurodevelopmental delay (Girchenko et al., 2020). In our study, the exposure of NSCs to medium conditioned with BeWo cells overexpressing *CYP11A1* showed not only significant cell cycle arrest in the G0 phase but also remarkable amount of double-strand DNA damage. These results may reveal the indirect effects of *CYP11A1* upregulation on fetal neurodevelopment (Figure 6).

Considering the unique pathway of vitamin D metabolism by P450scc, we explored the effect of this nutrient factor in cells overexpressing *CYP11A1*. Interestingly, vitamin D intervened in the signaling cascade and rescued the function of NSCs. This ability may be attributed to two mechanisms: competitive binding to excess P450scc with cholesterol and the anti-inflammatory and antioxidant effects of its product, 20(OH)D (Janjetovic et al., 2009; Janjetovic et al., 2010; Slominski et al., 2014; Slominski et al., 2015), the detailed mechanisms of which warrant further investigation.

To date, several clinical trials have reported the preventive effects or correlation of vitamin D supplementation in gestational diabetes, premature delivery, neurodevelopmental disorders, and preeclampsia during pregnancy, although some results remain controversial (Mojibian et al., 2015; Perez-Lopez et al., 2015; Fort et al., 2016; Zhang et al., 2016a; Zhou et al., 2017; Curtis et al., 2018). Combined with our findings, we presume that although the pathogeneses of these diseases have not been fully elucidated, trophoblast cell damage and inflammation caused by upregulation of *CYP11A1* expression might be involved and may be triggered by genetic factors or intrauterine exposure to adverse factors. It is assumed that vitamin D would be effective in treating these cases, but not in cases where *CYP11A1* upregulation is not involved. Additionally, vitamin supplement dosage is an important factor, indicating that population background, environment, diet, and even vitamin D receptor genotypes should be considered for precise and individualized supplementation (Ferrari et al., 1998; Niimi et al., 2000; Touvier et al., 2015).

## Conclusion

In summary, we elucidated the underlying mechanism of trophoblast damage caused by the upregulation of *CYP11A1*, which could be induced by toxins, drugs, hormones, or genetic variants. Furthermore, this upregulation of *CYP11A1* induced trophoblast damage impaired NSCs *in vitro*. If extrapolated to a clinical situation, these effects may lead to neurodevelopmental disorders. Furthermore, we found that vitamin D supplementation was an efficient intervention method to rescue the impairment described above and provided additional evidence to support vitamin D supplementation during pregnancy for the prevention of diseases.

Our *in vitro* study has limitations for extrapolating cell line results to primary trophoblast cells or *in vivo* studies. In particular, the mechanism of NSC damage requires further investigation. Considering the major structural and functional differences in the placenta between human and rodent models, the “human placenta-on-a-chip” device may provide an important model for future studies (Cox et al., 2009; Robbins et al., 2010; Schmidt et al., 2015; Blundell et al., 2018).

## Methods

### BeWo cell line culture and *CYP11A1* overexpression

BeWo cells (CCL-98, ATCC) were cultured in F12K medium (21127022, Gibco) containing 12% fetal bovine serum (FBS, 10270106, Gibco) and 1% penicillin/streptomycin (SV30010, Hyclone). Cells were divided into three groups: group 1 (CYP) was transfected with *CYP11A1* (NM_000781) overexpression lentivirus (GENECHEM) with a multiplicity of infection (MOI) of 25, group 2 (vehicle) was transfected with the equivalent negative lentivirus as a vector, and group 3 was not transfected and was used as the control (CTL). After green fluorescent protein (GFP) was expressed, puromycin (2.5 μg/ml final concentration) was added to select the stable cell clones*,* and the expression of *CYP11A1* at the mRNA and protein level was analyzed using qPCR and western blotting, respectively.

### Effects of *CYP11A1* overexpression on BeWo cells

After overexpression of *CYP11A1*, BeWo cell syncytialization was induced by the addition of 25 μM forskolin (F127328, Aladdin) for 48 h. The medium was then replaced with neuronal maintenance medium (NMM), containing 50% DMEM/F12 GlutaMax (31331028, Gibco), 50% neurobasal medium (21103049, Gibco), 0.5% GlutaMAX supplement (35050038, Gibco), 1 × B27 supplement (17504044, Gibco), 1 × N2 supplement (17502048, Gibco), 1% penicillin/streptomycin (SV30010, Hyclone), 10 ng/ml b-FGF (PHG0263, Gibco), and 10 ng/ml EGF (PHG0311, Gibco). Vitamin D_3_ was added at concentrations of 0, 2, or 5 μM to the BeWo culture of group CYP, whereas NMM plus the equivalent volumes of DMSO were added to the vehicle and CTL groups for 24 h.

The cell culture supernatant, named conditioned NMM were harvested, carefully centrifuged at 600 x *g* for 5 min, filtered through a 0.22 μm syringe filter to remove cells and debris, and stored at −80°C until further use. Then, the levels of secreted pregnenolone, progesterone, and inflammatory cytokines were analyzed.

Meanwhile, the treated BeWo cells were harvested to extract DNA, RNA, and protein. The expression levels of *PPARGC1A, NRF1, TFAM*, and *IL-6* were determined at the mRNA level using qPCR, and PGC-1α expression at the protein level was determined using western blotting. The level of mtDNA damage was assessed using qPCR, and mitochondrial ROS and apoptosis were assessed through MitoSox and JC-1 staining, respectively.

### Culture and exposure of NSCs to BeWo-conditioned medium

NSCs differentiated from human-induced pluripotent stem cells (hiPSCs) that were derived from human peripheral blood, were obtained from BioTalentum Ltd. The NSCs were seeded into laminin- (L2020, Sigma) and poly-L-ornithine- (P4957, Sigma) coated plates and cultured in NMM. After cell confluence reached 50%, the culture medium was replaced with conditioned NMM, as described above, or fresh NMM and cultured for 12 h. KI-67 immunofluorescence staining was performed to analyze NSC proliferation, and the results were confirmed using CCK-8 assays. γH2AX staining and immunofluorescence was performed to detect the amount of DSBs in the DNA.

### Antibodies

The antibodies used in the western blotting experiments were anti-CYP11A1 (1:200, 13363-1-AP, Proteintech), anti-PGC1α (1:200, AF5395, Affinity), anti-DDDDK Tag (1:1000, 66008-3-Ig, Proteintech), anti-GAPDH (1:5000, EM1101, Huabio), HRP-conjugated goat anti-mouse IgG (1:5000, ZB-2305, Zsbio), and HRP-conjugated goat anti-rabbit IgG (1:5000, ZB-2301, Zsbio). The antibodies used for the immunofluorescence experiments were anti-KI-67 (1:50, 27309-1-AP, Proteintech), anti-γH2AX (1:400, ab26350, Abcam), goat anti-mouse antibody, Alexa Fluor 594 (1:1000, A11005, Invitrogen), and goat anti-rabbit antibody, Alexa Fluor 594 (1:1000, A11012, Invitrogen).

### Western blotting

Cells were lysed using radioimmunoprecipitation assay (RIPA) buffer (P0013C, Beyotime) containing a protease and phosphatase inhibitor cocktail (B14011 and B15001, respectively, Bimake) to extract the total protein. Equal amounts of denatured protein samples were separated on 10% SDS-polyacrylamide gels and transferred to a polyvinylidene difluoride (PVDF) membrane (IPVH00010, Millipore) for immunoblot analysis. GAPDH was used as an internal control, and the intensity of bands was analyzed using Image J software (version 1.5).

### Staining and immunofluorescence

For JC-1 (C2006, Beyotime) and MitoSox (M36008, Invitrogen) staining, BeWo cells were cultured on glass coverslips and washed with 1 × Hank's balanced salt solution (HBSS) (14065056, Gibco). JC-1 or MitoSox was loaded onto the cells and incubated for 15 min at 37°C, protected from light. The cells were then washed three times with warm 1 × HBSS and counterstained with 4,6-diamidino-2-phenylindole (DAPI, P0131, Beyotime) to label the nuclei. At least five quality images were acquired using a laser scanning confocal microscope (Olympus FV2000).

For immunofluorescence, NSCs were cultured on glass coverslips, washed with Dulbecco’s phosphate-buffered saline (DPBS) (14190144, Gibco), fixed with 4% paraformaldehyde, permeabilized with 0.3% Triton X-100 (T8200, Solarbio), blocked with 5% bovine serum albumin (BSA, 4240GR500, Biofroxx) in PBS, and incubated with primary antibodies overnight at 4°C. Then, the slides were washed in PBS, incubated with Alexa Fluor 594-labeled secondary antibodies for 1 h at room temperature, and counterstained with DAPI to label the nuclei. At least five quality images were acquired using a laser scanning confocal microscope (Olympus FV2000).

### CCK-8 cell proliferation assay

NSC proliferation was measured using the CCK-8 (Abmole, M4839) assay. NSCs were seeded into laminin- and poly-L-ornithine-coated 96-well plates at a density of 5,000 cells per well and cultured with NMM for 24 h. The medium was then replaced with fresh or BeWo-conditioned NMM, as described above, and cultured further for 12 h. Then, 10 μl of CCK-8 solution was added to each well and incubated for another 4 h at 37°C. Absorbance was measured at 450 nm to determine cell viability, using a microplate reader (Cmax Plus, Molecular Devices).

### Quantitative analysis of secreted molecules

BeWo-conditioned NMM, as described above, was collected, and stored at −80°C until further use. Pregnenolone levels were determined using an ELISA kit (FR E-2700, LDN) and readout wavelength of 450 nm on a microplate reader (Cmax Plus, Molecular Devices). Progesterone analysis was performed in-house, using the ADVIA Centaur XP Immunoassay System (Siemens, Germany). MDA levels were assessed using an assay kit (A003-1-2, Nanjing Jiancheng) based on thiobarbituric acid (TBA). IL-1β, IL-4, IL-6, IL-8, IL-10, IL-13, IL-17α, IL-23, and TNF-α levels were analyzed using a suspension array (MAGPX12157001, Luminex). IL-6 levels were further verified by using an ELISA kit (E-EL-H0102, Elabscience) and readout wavelength of 450 nm on a microplate reader (Cmax Plus, Molecular Devices). All analyses were performed according to the manufacturers’ instructions.

### Quantitative PCR

BeWo cells were cultured and treated as described earlier, and total RNA was extracted using TRIzol reagent (Invitrogen, 15596026). A NanoDrop 2000 (Thermo Scientific) was used to measure the amount of total RNA in each sample, and 500 ng of total RNA was subjected to reverse transcription (RT) using an RT reagent kit (Takara, RR037A) to form cDNA. Quantitative PCR for cDNA was performed in triplicate using SYBR Green qPCR Master Mix (Bimake, B21202) on an iCycler RT-PCR detection system (Bio-Rad Laboratories). *GAPDH* was used as an internal control. Delta-delta Ct value analysis was used to evaluate the relative expression of target genes. Primer pairs were designed using Primer3plus (http://www.bioinformatics.nl/cgi-bin/primer3plus/primer3plus.cgi) and are listed in Table 1.

**TABLE 1:** Primer pairs used in qPCR.

Primers	Sequence	Product size
*CYP11A1*	F 5′−GGA​AAT​TAC​TCG​GGG​GAC​AT−3′ R 5′−TCG​GGG​TTC​ACT​ACT​TCC​TC−3′	113 bp
*TFAM*	F 5′– CCG​AGG​TGG​TTT​TCA​TCT​GT−3′ R 5′−ACG​CTG​GGC​AAT​TCT​TCT​AA−3′	147 bp
*NRF1*	F 5′−AAA​TGT​CCG​GAG​TGA​TGT​CC−3′ R 5′−CTG​TGT​TTG​CGT​TTG​CTG​AT−3′	148 bp
*PPARGC1A*	F 5′−GCT​GAC​AGA​TGG​AGA​CGT​GA−3′ R 5′−TAG​CTG​AGT​GTT​GGC​TGG​TG−3′	136 bp
*IL6*	F 5′−AAA​GAG​GCA​CTG​GCA​GAA​AA−3′ R 5′−AGC​TCT​GGC​TTG​TTC​CTC​AC−3′	183 bp
*GAPDH*	F 5′−ATG​TTC​GTC​ATG​GGT​GTG​AA−3′ R 5′−GTC​TTC​TGG​GTG​GCA​GTG​AT−3′	173 bp

### Evaluation of mtDNA damage

BeWo cells were cultured and treated as described earlier. Total genomic DNA was extracted using a specialized DNA extraction kit (DP304-03, TIANGEN) according to the manufacturer’s instructions. Treatment of normal BeWo cells with 500 μM H_2_O_2_ for 30 min was used as a positive control.

**FIGURE 7:**
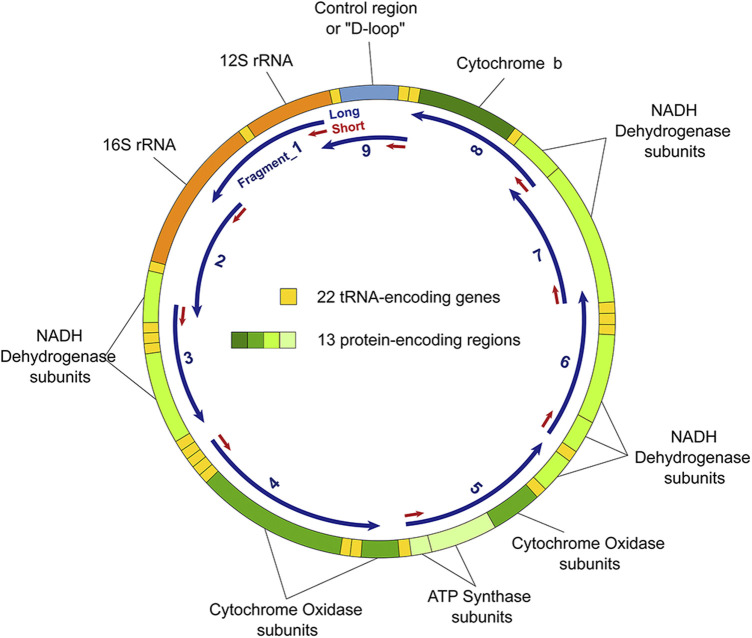
Mitochondrial DNA (mtDNA) and fragments used to estimate mtDNA damage. Fragment_1, ChrM: 298–2036; Fragment_2, ChrM: 2078–3403; Fragment_3, ChrM: 3318–4992; Fragment_4, ChrM: 5784–7517; Fragment_5, ChrM: 7858–9763; Fragment_6, ChrM: 9895–11856; Fragment_7, ChrM: 11775–13717; Fragment_8, ChrM: 13650–15381; and Fragment_9, ChrM: 15361–369.

According to the method of Oliver (Rothfuss et al., 2010) and Artem et al. (Gureev et al., 2017), mtDNA damage was estimated based on semi-long run qPCR. Briefly, 9 primer pairs were used to amplify 9 long mtDNA fragments, ranging in size from 1,300 to 2,400 bp, whereas another pair of primers was used to amplify a short fragment ranging in size from 68 to 140 bp, located at the beginning of each long fragment (Figure 7). We used the primer pairs previously described by Artem et al. (Gureev et al., 2017) and listed in Table 2. For amplification, 5 ng of total DNA was utilized to perform qPCR using SYBR Green qPCR Master Mix (Bimake, B21202) on an iCycler RT-PCR Detection System (Bio-Rad Laboratories). The reaction conditions were: pre-incubation for 5 min at 95°C followed by 40 cycles of 95°C for 10 s, 61°C for 30 s, and 72°C for 10 s (small fragments) or 90–120 s (long fragments).

**Table 2:** Primer pairs used for mtDNA damage analysis.

Primers	Sequence	Product size
Fragment_1	F 5′−TAA​ATT​TCG​TGC​CAG​CCA​CC−3′ R 5′−GTT​GAC​ACG​TTT​TAC​GCC​GA−3′ (Short) R 5′−ATG​CTA​CCT​TTG​CAC​GGT​CA−3′ (Long)	
		72 bp
		1739 bp
Fragment_2	F 5′−ACG​AGG​GTC​CAA​CTG​TCT​CTT​A−3′ R 5′−AGC​TCC​ATA​GGG​TCT​TCT​CGT−3′ (Short) R 5′−CCG​GCT​GCG​TAT​TCT​ACG​TT−3′ (Long)	
		97 bp
		1326 bp
Fragment_3	F 5′−CTA​GCA​GAA​ACA​AAC​CGG​GC−3′ R 5′−CCG​GCT​GCG​TAT​TCT​ACG​TT−3′ (Short) R 5′−TTA​GGG​CTT​TGA​AGG​CTC​GC−3′ (Long)	
		86 bp
		1675 bp
Fragment_4	F 5′−GGC​GGT​AGA​AGT​CTT​AGT​AGA​GAT−3′ R 5′−TGG​CTG​AGT​AAG​CAT​TAG​ACT​GT−3′ (Short) R 5′−CTA​GGG​AGG​GGA​CTG​CTC​AT−3′ (Long)	
		136 bp
		2334 bp
Fragment_5	F 5′−AAC​ATT​CCC​ACT​GGC​ACC​TT−3′ R 5′−TGT​TGG​GGT​AAT​GAA​TGA​GGC​A−3′ (Short) R 5′−TTG​TGT​TCA​TTC​ATA​TGC​TAG​GC−3′ (Long)	
		108 bp
		1925 bp
Fragment_6	F 5′−ACC​TCA​CCA​TAG​CCT​TCT​CAC−3′ R 5′−TGC​CTT​CCA​GGC​ATA​GTA​ATG​T−3′ (Short) R 5′−ATG​TGG​TGG​TGT​ACA​GTG​GG−3′ (Long)	
		87 bp
		1960 bp
Fragment_7	F 5′−TCA​TTC​TTC​TAC​TAT​CCC​CAA​TCC−3′ R 5′−ATG​TGG​TGG​TGT​ACA​GTG​GG−3′ (Short) R 5′−TGG​TTT​GGG​AGA​TTG​GTT​GAT​G−3′ (Long)	
		81 bp
		1942 bp
Fragment_8	F 5′−CCC​CAA​TCC​CTC​CTT​CCA​AC−3′ R 5′−TGG​TTT​GGG​AGA​TTG​GTT​GAT​G−3′ (Short) R 5′−GGT​GGG​GAG​TAG​CTC​CTT​CTT−3′ (Long)	
		68 bp
		1732 bp
Fragment_9	F 5′−AAG​AAG​GAG​CTA​CTC​CCC​ACC−3′ R 5′−AGC​TTA​TAT​GCT​TGG​GGA​AAA​TAG​T−3′ (Short) R 5′−GTT​GAC​ACG​TTT​TAC​GCC​GA−3′ (Long)	
		139 bp
		1308 bp

To calculate the DNA lesions in each long fragment, delta Ct (every other group versus CTL) of the long fragment (Δ_long_) was compared to the delta Ct of its corresponding short fragment (Δ_short_):

$$Lesion\;\;per\;\;10\;kb=\left(1-2^{-\left(\text{Δlong}-\text{Δshort}\right)}\right)\times \frac{10,000\;\;bp}{fragment\;\;size}$$

### Statistical Analysis

Data were analyzed using GraphPad Prism software package (version 8.4; La Jolla, United States) and are presented as Mean ± SEM. One-way ANOVA test was used to assess differences among more than two groups and Tukey’s post hoc test was used to determine the difference between each two groups. A *P*-value <0.05 was considered statistically significant.

## Data Availability

The raw data supporting the conclusions of this article will be made available by the authors, without undue reservation.
