# Supplementary figures and images for: CYP11A1 Upregulation Leads to Trophoblast Oxidative Stress and Fetal Neurodevelopmental Toxicity That can be Rescued by Vitamin D
Source: Front Mol Biosci. 2021 Feb 15;7:608447. doi: 10.3389/fmolb.2020.608447 (PMC7917044; doi:10.3389/fmolb.2020.608447)

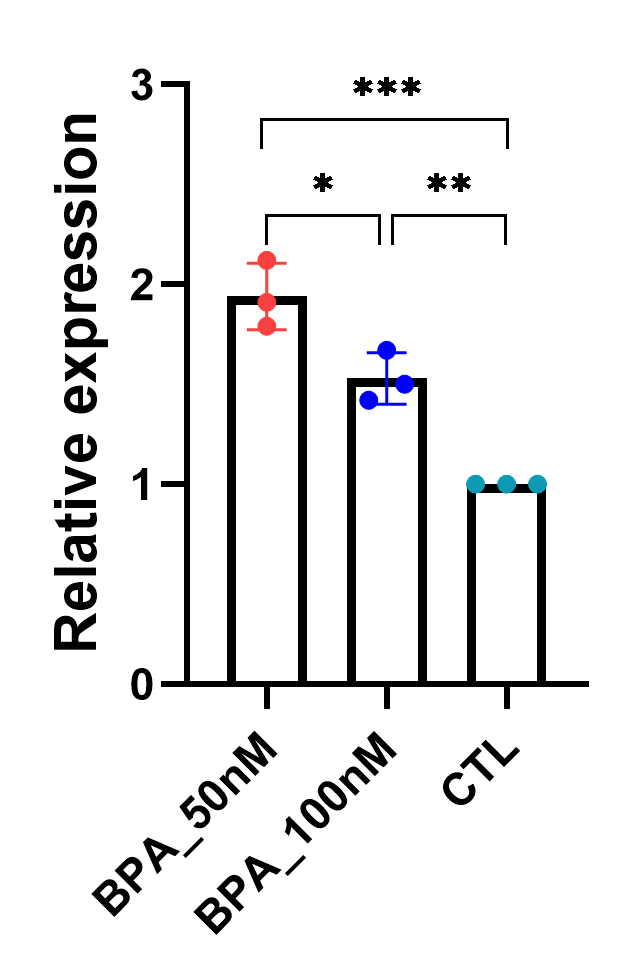

Supplement: Supplementary file 1 [file image1.tif]
